# Association of Workplace Bullying and Workplace Vulnerability in the Psychological Distress of Chilean Workers

**DOI:** 10.3390/ijerph16204039

**Published:** 2019-10-22

**Authors:** Elisa Ansoleaga, Magdalena Ahumada, Andrés González-Santa Cruz

**Affiliations:** 1 Faculty of Psychology, Diego Portales University, Santiago 7630103, Chile; 2 Institute of Public Health of Chile, Santiago 7780050, Chile; 3 Faculty of Social Sciences and History, Diego Portales University, Santiago 8370127, Chile

**Keywords:** workplace bullying, Chile, workplace vulnerability, psychological distress

## Abstract

Workplace bullying has been identified as a global problem because of its growing magnitude and the harmful effects in victims and organizations. Workplace vulnerability is a component of job precarious1ness that reflects insecurity, fear, and labor uncertainty. This paper aims to analyze the associations between the exposure to workplace vulnerability and psychological distress, and to explore the associations between exposure to workplace bullying and psychological distress, by sex. A cross-sectional and probabilistic survey was applied to a randomly-selected valid sample of 1995 salaried workers in three main metropolitan areas of Chile. Chi-square test and logistic regression models controlling for confounders were tested. Female workers were more exposed to workplace vulnerability and presented a higher prevalence of psychological distress. Among women who were vulnerable, one of three reported psychological distress (30.8%), which is higher than men (16.5%). Workers exposed to workplace vulnerability had a greater chance of workplace bullying, workers who perceived high workplace vulnerability had a greater chance of psychological distress, and workers exposed to workplace violence had a greater likelihood of psychological distress in comparison to those who were not exposed. Increasing employment security can reduce the perception of job vulnerability and help prevent the existence of workplace bullying. Additionally, occupational health protection policies should prevent, protect from, and intervene in workplace bullying as a precursor to mental health problems in Chile.

## 1. Introduction

Workplace bullying has been identified as a global problem because of its growing magnitude and the harmful effects it has in its victims. 

There are three main conceptual models of workplace bullying: an initial model, which emphasizes the individual characteristics of the aggressor and the victim, a second model, which examines the characteristics of the interactions between aggressor and victim, and the third model, which emphasizes social and organizational dimensions and culture [1]. Regarding the organizational dimensions, this last approach, proposed by Einarsen et al., has included psychosocial factors in the emergence of workplace bullying. Among others factors, it highlights: quantitative overload, emotional demands, work control and supervision systems, reward systems, and leadership styles [1,2,3]. Regarding social dimensions, an enabling condition of workplace bullying is the power imbalance between employees and superiors—including supervisors—which could also increase due to the lack of job stability and security [4].

Along with these aspects, the influence of social and economic context has been proposed as a relevant factor in the study of workplace violence, as violence can be considered a social phenomenon. Within the framework of the globalized economy, employment is undergoing important processes of change, including the expansion of service work and the generalization of working conditions, as characterized by: a variety of atypical contractual forms, work structures, and schedules in which the flexibility and precariousness of the employment predominate, which may lead to low incomes, restricted or no social protection, the limited-duration or complete absence of contracts, and limited possibilities for union organization and collective negotiation [5]. These conditions affect the worker on a daily basis, generating reactions such as fear of exclusion, violence, and lack of recognition: in short, a perception of vulnerability that further weakens the possibility of organization among workers [6].

The perception of vulnerability has been described in different ways: insecurity, fear, precariousness, and labor uncertainty, among other terms. Authors such as Benach and Juliá and colleagues conceive vulnerability as a component of job precariousness that is reflected in the perception of oneself as helpless in the face of unacceptable practices in the workplace, in addition to feeling incapable of asserting one’s rights, and in terms of the greater susceptibility to suffer by the actions (or non-actions) of employers [7,8]. However, it also contemplates the perception of one’s inability to maintain a desired continuity in a job and its conditions, much like the conceptualization of subjective job insecurity [8]. Other authors indicate that the perception of workplace vulnerability is related to labor precariousness, such as type of occupation and type of contract, along with macroeconomic measures such as the unemployment rate [9]. In turn, workplace vulnerability is associated with low job satisfaction and mental health [10]. Among the possible explanations of this association, authors postulate that precarious jobs imply greater flexibility, but less opportunities for socialization and less time for conflict resolution [11]. Similarly, job insecurity, a component of the perception of vulnerability, not only increases the ability to tolerate abusive behavior, but can also be associated with frustration that may lead to harassment at work [12,13]. It has also been shown that economic vulnerability, understood as a hazardous position in the labor market and for income insecurity, would produce mostly detrimental reactions to the perception of job security [10].

Added to this, vulnerable employments also permeate the social bonding of workers, giving rise to interactions where violence predominates within the workplace [12]. In this regard, it has been shown that violent behavior within work teams can be considered as aggressive actions toward a less powerful member of the group, especially when the direct source of frustration may be too dangerous [13]. Thus, multiple effects of workplace vulnerability have been observed in workplace violence, as well as in workers’ mental and physical health [11,14,15], and in work performance [14].

However, the effects of vulnerability at work are not randomly distributed among the working population, but instead they affect specific groups of workers with less formal or social power in organizations, such as those with lower qualifications and women. In regard to women, an explanation of workplace vulnerability is related to the model of labor flexibilization, which reproduces hierarchical relations that subordinate women, leading to their greater vulnerability to workplace violence [15]. As stated above, it has been observed that forms of destructive leadership are considered as a manifestation and consequence of inequalities within the workplace [16]. Moreover, the work-family conciliation would contribute to vulnerable employment conditions for women, in comparison with their male peers [16]. Among the consequences of workplace bullying are those that affect individuals, such as stress reactions, substance abuse, sleep problems, and mental and general health problems [1,17]. Likewise, workplace violence would also have consequences at the organizational level, impacting upon absenteeism rates, rotation, and productivity, among others [1]. It has also been stated that constructions about gender would influence the degree of exposure to bullying, as well as its consequences, which would vary according to sex [5]. 

**Figure 1:**
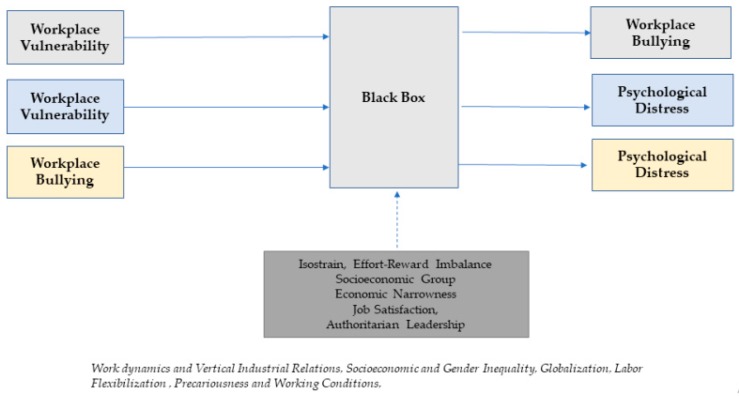
Theoretical framework by sex.

In fact, as indicated by Karen Messing, the consideration of a gender perspective in occupational health is very important because women are exposed to different labor risks from men, as they occupy different posts, and have less formal and social power. Hence, the relevance of considering men and women as different labor populations, which is included in the social structure [18,19]. A framework that show the dynamics of this process is proposed in Figure 1.

### 1.1. Chilean Work Context

Chile has almost eighteen million inhabitants, of which half are women and half are men. The workforce in Chile is 8,600,000, with salary earners representing seventy-one percent of the employed population. The workforce participation rate in 2015 was 82.9% for males and 54.9% for females [20].

Chile is a very unequal society; the per capita income of the richest 1% is forty-times higher than the per capita income of the remaining population. The labor scenario in Chile is characterized by the influence of flexibilization processes, with a growing variety of forms of employment [21,22]. In recent years, there has been an increase of part-time employment, informal employment, subcontracts of women, and lack of social security protection [23], as also noted through the National Employment Survey on Work, Health and Quality of Life of Workers in Chile [*Encuesta Nacional De Empleo, Trabajo, Salud Y Calidad De Vida De Los Trabajadores Y Trabajadoras En Chile*] (ENETS) applied in 2009. There is a generalized feeling of vulnerability, and women often have insecure working conditions [23].

Rodríguez and Gómez [24] point out that the main elements of work dynamics in Chilean companies are: the existence of great pressure exerted by managers and senior officials focused on organizational efficiency, and a lack of workers’ autonomy and empowerment. Moreover, loyalty, dedication, fulfillment, and professionalism are desired attributes in workers. Meller points out that the pressure for work and the focus on efficiency are particularly descriptive of the prevailing discourse adopted by Chilean employers [24]. In this context, organizations are geared toward maximizing profits with little regard for workers and their well-being. On the other hand, the Organization for Economic Cooperation and Development (OECD) stated that industrial relations in Chile are generally confrontational and marked by a lack of trust [25]. According to the agency, the conflict is partly due to limited union coverage. The permanence of this model entails a serious breach of the basic standards of efficacy and of workers’ participation in the exercise of their rights. Berghuis adds that the “neoliberal reforms in Chile exemplify economic violence because the conditions lead to women being discriminated for being worth less than men in the work field, and decreasing their access to services such as healthcare, additionally it can be categorized as structural violence as the state was the initiator of the policies creating a society in which women are disadvantaged” [26].

### 1.2. The Problem to be Addressed

Despite the previous antecedents, in Chile much of the studies carried out so far on workplace bullying were conducted on small samples of target populations and focused on identifying the various forms of expression of workplace violence [27]. These studies have provided a history of the persons or groups most vulnerable to violence, the facilitators or promoters of violence, victims’ ways of coping with them, and their effects at the level of individuals, work groups, and businesses, among other topics addressed. However, until now, a study of the magnitude of this work, which would estimate the prevalence of workplace bullying in the general working population and consider its relationship with mental health, including an analysis of the social and gender inequalities involved in the labor market, had not been carried out in Chile.

The contribution of this work, on the one hand, is that it provides evidence of the exposure to workplace violence in Chile, analyzing its relationship with mental health in the general population, and estimating the magnitude of the phenomenon, and, on the other hand, the novelty of this study is that it includes the dimensions of social inequalities by incorporating workplace vulnerability and social inequalities into a sex-stratified analysis of workplace bullying and mental health. 

The objective of this article is to identify associations between exposure to workplace bullying and the perception of workplace vulnerability in participants, to analyze the associations between exposure to workplace vulnerability and the psychological distress of the participants, and to explore the associations between exposure to workplace bullying and psychological distress according to sex.

The following hypotheses guided this work: (a) Workers exposed to workplace vulnerability have a greater likelihood of reporting being a victim of workplace bullying in comparison to those who are not exposed. (b) Workers exposed to work vulnerability have a greater likelihood of presenting psychological distress compared to those who are not exposed. (c) Workers exposed to workplace bullying have a greater likelihood of presenting psychological distress. All the above also take into account control variables and gender heterogeneity.

## 2. Materials and Methods 

This study was approved by the research ethics committee of Diego Portales University. All participants signed a consent form in which they were informed of the objectives of the study and the safeguarding of their rights and ethical aspects. 

### 2.1. Sample

Within the framework of the project FONDECYT Regular N° 1170239, the survey was applied between March and May 2018 to a random sample of salaried workers in three main metropolitan areas of the country (Gran Santiago, Gran Valparaíso, and Gran Concepción). From the 2000 people eligible to answer the survey the response rate was 87,3%, with 1995 valid and suitable for analysis cases. The design is cross-sectional and probabilistic and the sample was randomly stratified and selected in three stages (district, household, and individual). Women workers were over-represented in order to obtain an equivalent sex ratio. Subsequently, the information was weighted based on data from the National Employment Survey [*Encuesta Nacional de Empleo*] (ENE), updated to the first quarter of 2018, in regard to the occupational proportion by sex and region. An average of 971 men (49%) and 1024 women (51%), aged between 43 and 41 years, respectively, were surveyed in the metropolitan locations of Santiago (40%), Concepción (30%), and Valparaíso (30%), respectively. About 82% were in the private sector. Less than one-third were supervisors (29%). A classification by occupation shows that most respondents were unskilled workers (19%), operators (16%), office employees (12%), sales persons (11%), service workers (11%), intermediate managers (8%), professionals (8%), technicians (8%), and transport drivers (6%). In addition, more than half (59%) worked in organizations with more than 50 employees. 

### 2.2. Variables

#### 2.2.1. Under Study

Workplace Vulnerability: The scale used is part of an adaptation of the Vulnerability subscale, consisting of 4 items from the Precarious Employment Scale (EPRES) as developed and validated in the Chilean context by Vives–Vergara and colleagues [26,28], plus 3 items from the Insecurity sub-dimension with respect to the work contract, from the instrument SUSESO-ISTAS 21 [29]. The latter was used in the ENETS Survey and obtained good internal consistency (α = 0.88) [30]. The instrument had adequate internal consistency in this sample (α = 0.85). The score was calculated from the sum of the different indicators, similar to the score used in other studies [26]. Subsequently, the cases were categorized as situations of perceived vulnerability when the respondents indicated that they suffered three or more of the seven elements present in the scale: fear of demanding better working conditions, feeling of unfair treatment, concern about changing conditions, fear of dismissal if they do not do what they are asked to do, concern about dismissal or non-renewal, concern about the difficulty of finding another job in the event of dismissal, and the feeling that one can be easily replaced (for e.g., “Afraid to demand better working conditions”).

#### 2.2.2. Covariates

Negative Acts Questionnaire (NAQ-R): Composed of 22 sentences in which the respondent must indicate the frequency of exposure to behaviors related to bullying in the workplace in the last six months (for e.g., “Being shouted at or being the target of spontaneous anger”) on a five-point Likert scale ranging from "Never" (0) to "Daily" (4), formulated in behavioral terms and that do not mention the word “harassment” or similar words. The Anglo–American version of the instrument [31] was translated by González and Graña [32]. The test showed a very high internal consistency (α = 0.93). The operational criteria proposed by Mikkelsen and Einarsen categorizes as victims individuals who were exposed to at least two behaviors with a frequency greater than "Weekly" [33].

Psychological Distress (K6) [34]: Composed by six items related to the prevalence of six symptoms related to depression and anxiety (for e.g. “Nervous”, “Restless”, etc.) during the past thirty days, ranging from “Always” (4) to “Never” (0). This measure was validated in a Chilean sample by Ansoleaga and colleagues [34]. The test showed a high internal consistency (α = 0.85). The sum of the scores was recoded as high distress if the respondent scored seven or higher.

The ISOSTRAIN variable (high job demands plus low decisional latitude and low social support in the workplace) comes from Karasek and Theorell’s model and was measured by the items of the Work Content Questionnaire [35], selecting those respondents who simultaneously present high psychological demands (5 original items of the scale plus 4 of the emotional overload subscale of the SUSESO-ISTAS 21 instrument), low decisional latitude (5 items), and low social support from superiors or peers (6 items). This measure was validated in a Chilean sample by Ansoleaga and colleagues. The test showed an acceptable internal consistency (α = 0.79) [34].

Effort-Reward Imbalance (ERI) [36] is composed of two subscales: Effort (3 items) in the number of tasks, work pace, and interruptions experienced; and Rewards (7 items), consisting of questions related to esteem, promotion possibilities, and job stability. Imbalance is the result of a combination of greater effort and low-perceived rewards. This measure was validated in a Chilean sample by Ansoleaga and colleagues [34]. The test showed an acceptable internal consistency (α = 0.68).

Job Satisfaction, a variable linked to leadership styles in theoretical and empirical models, was measured through 5 items from the job satisfaction scale from Judge and collaborators (for e.g., “Most days I am enthusiastic about my work”). Responses were evaluated on a “strongly disagree” (1) to “strongly agree” (4) scale. [37,38]. Job satisfaction was conceived as the sum of its components [37]. It was recoded to account for low job satisfaction, with the cut-off point in the second quartile of the sum of raw scores. The test showed an acceptable internal consistency (α = 0.73).

Authoritarian Leadership [39], measured with four items of the Destructive Leadership Scale, in which respondents indicate how often their supervisor has performed behavior favoring the organization but at the expense of the employees (for e.g., “Has been chummy by encouraging you/your colleagues to extend your/their lunch break.”), ranging from “Never” (0) to “Almost Always” (3). The test showed an acceptable internal consistency (α = 0.70). The criterion defined by Leymann [40] was used to classify those exposed to this leadership style (those who report at least one behavior with a frequency of "Quite often"(2)).

Among other socioeconomic characteristics, Economic Narrowness was calculated with an item in which the respondents are asked the extent to which their monthly household income is sufficient to support their basic needs. The cases where the income was insufficient were recoded.

Another socioeconomic measure is the Socioeconomic Group of the respondent, which was calculated in order to determine the socioeconomic groups D and E, which are the groups with lower incomes. This classification was elaborated by the Adimark company (currently, GFK) from the information provided by the Chilean census applied in 2002, which uses among its indicators the monthly household income and the educational level of the head of household.

### 2.3. Data Analysis 

In order to identify gender differences, all the analyses described below were segmented by sex. A chi-square independence test was carried out, with the Rao–Scott adjustment for complex samples, to determine possible relationships between the categories of different dichotomous variables [41] in the Stata software. Subsequently, different weighted logistic regression models were tested. For each sex, we determined the likelihood of suffering workplace violence in workers who perceive workplace vulnerability and the likelihood of presenting psychological distress in workers exposed both to workplace vulnerability and workplace violence. Both of these variables were operationalized according to the criterion of Mikkelsen and Einarsen [33]. Raw analyses were performed and subsequently adjusted for variables that the literature has identified as possible confounders. In case of the association between workplace violence and psychological distress, workplace vulnerability was included as a confounder in every model. Two main control covariates were established concerning psychosocial variables related to the organization of work (effort–rewards imbalance and ISOSTRAIN); two covariates from the destructive leadership theory (Authoritarian Leadership and Low Job Satisfaction), and two covariates from other socioeconomic characteristics (Economic Narrowness and Socioeconomic Groups D and E). The coefficients obtained were translated into probabilities or odds ratio (OR) and their confidence intervals (CI), in order to compare the risks as a function of gender when controlling for the mentioned covariates [42].

## 3. Results

The prevalence of workplace bullying following the criteria provided by Mikkelsen and Einarsen was of 9.8% among female workers, and 10.5% among male workers; 39.7% of female workers perceived workplace vulnerability, while 34% of male workers perceived workplace vulnerability; 18.2% of female workers reported psychological distress, while 9.6% of male workers reported psychological distress.

### 3.1. Bivariate Association 

Overall, 36.4% (40% of women and 34% of men) of respondents felt vulnerable at their workplace. In Table 1, it can be seen that all covariates showed an association with workplace vulnerability, with the exception of Economic Narrowness, which did not show significant differences in any of the sexes. At a descriptive level, more than half of the sample studied in a situation of vulnerability perceived an imbalance between the efforts made at work and the rewards received (women = 65.4%; men = 58.9%). At the same time, nearly 4 out of 10 vulnerable respondents reported low job satisfaction, with a higher proportion for women (41.3%) than for men (34.6%). Similarly, about four out of ten vulnerable respondents reported economic narrowness, with a higher proportion among women (40.9%) than among men (35.7%). Finally, among vulnerable women workers, one out of three reported psychological distress (30.8%) compared to male workers in the same situation (16.5%).

### 3.2. Multivariate Analysis

In order to study the associations between exposure to vulnerability and workplace violence and distress or between workplace violence and psychological distress while controlling for workplace vulnerability, multivariate analyses were carried out incorporating variables that would influence these associations, such as psychosocial factors of work, work characteristics of the destructive leadership theory, and other socioeconomic characteristics.

As can be observed in the raw analyses in Table 2, in women, exposure to workplace vulnerability increases the probability of reporting workplace bullying, which is reflected in odds ratios greater than 1 (OR^raw^ = 3.29 [1.98–5.49]), as well as psychological distress (OR^raw^ = 4.16 [2.74–6.31]), and to a lesser extent, suffering workplace bullying increases the likelihood of reporting psychological distress (OR^raw^ = 2.28 [1.35–3.88]). When controlling for the psychosocial dimensions of the organization of work, the effect of exposure to workplace vulnerability decreased (OR^1^ = 1.97 [1.07–3.64]), but its statistical significance is maintained. On the other hand, the effect was lower albeit significant when controlling for labor variables linked to authoritarian leadership and job dissatisfaction (OR^2^= 2.21 [1.30–3.76]), except for the effect of workplace violence on psychological distress, also controlling for workplace vulnerability, where no significant effects were observed (OR^2^ = 1.44; 95% CI [0.77–2.68]). On the other hand, when controlling for the socioeconomic variables, the magnitude of the effect shown in the raw model decreased slightly although it maintained an important statistical significance (*p* < 0.01), both in the effect of workplace vulnerability on the likelihood of reporting workplace violence (OR^3^ = 3.50; 95% CI [2.10–5.86]) and psychological distress (OR^3^ = 4,02; 95% CI [2.62–6.17]), as well as the effect of workplace bullying on psychological distress, also controlling for workplace vulnerability (OR^3^ = 2.13; 95% CI [1.24–3.65]).

Based on the analysis of the male sample segment, exposure to workplace vulnerability increase the odds of reporting workplace violence (OR^raw^ = 2.32; 95% CI [1.46–3.69]), as well as of reporting psychological distress (OR^raw^ = 2.95; 95% CI [1.78–4.89]), just as exposure to workplace violence increased the likelihood of presenting psychological distress when controlling for workplace vulnerability only (OR^raw^ = 4.27; 95% CI [2.33–7.86]), in raw analyses. Male workers exposed to workplace violence had at least 2.39 times (95% CI [1.20–4.77]) more likelihood of presenting psychological distress, even when controlling for the psychosocial variables of work organization and labor related to leadership, although the effect of workplace violence on psychological distress when controlling for the socioeconomic variables (OR^3^ = 4.01; 95% CI [2.16–7.46]) is particularly important. On the other hand, the effect of workplace vulnerability among males tended to decrease significantly, until losing significance after controlling for other variables such as the psychosocial and labor variables linked to leadership (OR^1^ = 1.43 [.87–2.35] and OR^2^ = 1.56 [.95–2.58]), excepting socioeconomic variables (OR^3^ = 2.23 [1.38–3.61]) (see Table 2).

When comparing the effects of workplace vulnerability on workplace bullying between men and women (See Table 2), it can be seen that vulnerable women workers would be more likely to present both workplace violence (OR OR^raw^ in women = 3.29 vs. OR^raw^ in men = 2.32) or psychological distress (OR^raw^ in women = 4.16 vs. OR^raw^ in men = 2.95), without controlling for other variables. In contrast to the significant effects observed in female workers, the effect of workplace vulnerability on workplace violence in men loses statistical significance when controlling for psychosocial dimensions (OR^1^ in men = 1.43 vs. OR^1^ in women = 1.97), similarly when controlling for labor variables related to leadership (OR^2^ in men = 1.56 vs. OR^2^ in women = 2.21). On the other hand, the effect of workplace violence on the chance of presenting psychological distress is lower in women (OR^raw^ in men = 2.29 vs. OR^raw^ in women = 4.27). In fact, unlike the significant effects in men, the effect ceases to be significant in women when controlling for labor variables related to Leadership (OR^raw^ in women = 1.44 vs. OR^raw^ in men = 2.39).

## 4. Discussion

Regarding the hypotheses presented in this study and despite the existence of differences among sexes, it can be stated that workers exposed to workplace vulnerability have a greater likelihood of being victims of workplace bullying when compared to those who were not exposed; workers who perceived high workplace vulnerability had a greater likelihood of presenting psychological distress when compared with those who were not exposed; workers exposed to workplace violence had a greater likelihood of presenting psychological distress, without considering confounding elements; and workers exposed to workplace bullying had a greater likelihood of presenting psychological distress, only controlling for workplace vulnerability. However, when controlling for psychosocial risk factors (ISOSTRAIN, produced by the combination of high demands, low decision and no social support, and Effort–Rewards Imbalance) and labor variables related to destructive leadership, such as authoritarian leadership and low job satisfaction in men, the association between workplace vulnerability and workplace violence lost its statistical significance. Whereas, in women, the relationship between workplace bullying and psychological distress loses statistical significance when controlling for labor characteristics linked to destructive leadership.

The results in relation to the effects of workplace vulnerability on workplace bullying as well as on the psychological distress of workers are consistent with international evidence [1,14]. Female workers are more exposed to workplace bullying and workplace vulnerability, and present a higher prevalence of psychological distress. In this regard, it can be seen that, among women who were vulnerable, one of three reported psychological distress (30.8%), compared to men in the same situation (16.5%).

Workplace vulnerability shows an association with psychological distress in women, even when controlling for psychosocial, labor linked to leadership and socioeconomic variables. In short, women in workplace vulnerability suffered psychological distress [43]. These findings were consistent with similar studies indicating associations between the two variables [44,45,46,47,48,49]. Moreover, they could be framed among the documented gender differences in the workplace as determinants of mental health inequities [47]. In this regard, there is evidence that exposure to workplace bullying is directly related to symptoms of distress, affecting women’s well-being [43]. Furthermore, women who perceive discrimination at work tend to report more health problems, including distress, insecurity, and lack of autonomy [50]. Evidence also shows that vulnerable jobs are more likely to produce psychological distress, which increases in women [49], and that forms of destructive leadership are mainly related to situations of workplace vulnerability [51]. 

According to Roscigno, Hodson, and Lopez [52], workplace bullying occurs through the influence of certain organizational dynamics, generating disadvantageous scenarios for women in which supervisors adopt violent attitudes. This makes women even more vulnerable to victimization and to tolerate workplace bullying and its consequences [53].

At the same time, these results are related to the findings of a qualitative study applied in Chile, in which the workers mentioned that one of the manifestations of violence was being treated as "disposable" and not being recognized for their work [51]. Similarly, the results concerning exposure to workplace vulnerability and the higher probability of reporting workplace bullying in women also coincide with the results of the study applied in Chile, which found that, especially in retail sectors, characterized by poor employment conditions and a larger population of female workers, there is greater tolerance of arbitrariness and abuse, as well as less individual coping capacity [51]. The qualitative study was also consistent with the results of the Araucaria survey conducted at the beginning of this decade in Chile, which established gender differences in exposure to psychological and sexual harassment, and physical or verbal violence [54]. However, results not reported on this paper showed that, for men, the exposure to workplace bullying presented socio-occupational differences being higher for manual workers than for professionals, whereas for women, it was transversal. These results are relevant, as they coincide with the findings of other studies on workplace bullying; for example, that women have a higher risk of workplace bullying, especially when analyses are differentiated by industry (e.g., education sector), or organizational characteristics (e.g., work hours) [55]. This confirms the relevance of studying the differences in the exposure of men and women in occupational health, not only as a theoretical intention, but also because ignoring the differences can lead to errors when identifying risk and health problems, encouraging discriminatory practices [18,19].

In the present study, workplace vulnerability was associated with a greater chance of reporting workplace bullying both in men and women, although higher in women (OR^raw^ = 3.3 in women, vs. OR^raw^ = 2.3 in men), and this difference slightly increased when maintaining other socioeconomic characteristics constant (OR^3^ = 3.5 in women, vs. OR^3^ = 2.2 in men), whereas in men, the association between workplace vulnerability and workplace bullying did not exist when controlling for the psychosocial dimensions and work characteristics linked to destructive leadership.

Another element to consider is that, in women, the effect of workplace violence on psychological distress decreased and lost statistical significance, when incorporating the variables of authoritarian leadership and low job satisfaction. This could be explained by the fact that low job satisfaction could occur as a consequence of authoritarian leadership, and its effects could be perceived after six months at least of exposure to such behaviors [38]. Worth noting is that, in general, women could perceive authoritarian leaderships more frequently, which is also closely related to workplace bullying, distorting its effects [56]. This leads to questions about the origin of situations of the workplace bullying of women and its effects on the psychological distress of workers. 

This results are relevant in terms of the bindings between medical leave and job absenteeism for occupational causes, and mental illnesses, as the relationship between workplace bullying and absenteeism due to illness has been studied internationally [57], and specifically in Chile, where mental illnesses represent the most frequent diagnosis of occupational illness and account for the highest percentage of job absenteeism [23]. On the other hand, this finding could shed light on the protective characteristics of the promotion of constructive leaderships against workplace violence [58]. Finally, regarding the fact that more than half of the population studied who were in a situation of workplace vulnerability perceived an imbalance between the efforts made at work and the rewards received, it is necessary to consider that people who perceive this type of imbalance are also more likely to be exposed to workplace violence [51]

### Limitations

As the study design is cross-sectional, it is not possible to indicate empirical causality relationships. However, a large part of the hypotheses proposed are related to findings from longitudinal studies, which identify causal relationships through data, ruling out bidirectionality or reciprocity of the effects.

It is important to mention that gender differences may be more complicated than just the mere association of two variables, even when controlling for other variables [11]. In this sense, according to theoretical background, it could be hypothesized that greater workplace and socioeconomic vulnerability would influence workplace violence, which in turn would influence mental health, such as psychological distress [59]. Moreover, women’s exposure to workplace violence has been shown to be related to important differences in power and control at the workplace, causing significant damage to health and well-being, particularly in women.

In relation to the battery of instruments applied, as they were administered face-to-face, there could be methodological effects associated with social desirability, especially in topics that have a negative connotation, such as mental health indicators, workplace violence, and income [60].

On the other hand, the results cannot be generalized to a rural population or to more extreme regions of the country, because they were applied in metropolitan regions.

Another limitation has to do with the recent social changes in our country. We recommend as a challenge for future studies to delve into other aspects of workplace and social vulnerability, such as migratory status, linguistic barriers, ethnic differences, sexual preferences, gender identities, and among other minorities that in other countries have been unprotected against workplace harassment [61,62]

## 5. Conclusions

In conclusion, the results of this paper confirm the associations reported in the literature regarding workplace vulnerability and workplace bullying, and between these two factors and the psychological distress reported by the workers.

Workers who perceive a high vulnerability have a greater probability of presenting psychological distress when compared to those who perceive low vulnerability. Hence, there would be an association between workplace vulnerability and distress, still controlling for psycho-social and labor factors linked to destructive leadership and other socioeconomic characteristics.

These results, in turn, contribute to consolidating the hypothesis of the importance of the labor and macrosocial context to understand workplace bullying. Many studies have shown the relationship between job precariousness and workplace bullying. This study adds the workplace vulnerability component as a subjective dimension regarding employment conditions linked to the fear of losing one’s job or worsening working conditions. This study showed that workplace vulnerability is closely related to the emergence of workplace bullying and this, in turn, increases the probability of high or very high distress. It is therefore proposed to address the objective and subjective conditions of employment, precariousness, and workplace vulnerability, respectively, as relevant dimensions in the production of workplace violence. In fact, reducing the perception of job vulnerability and increasing employment stability and security can help prevent or limit the existence of workplace violence in Chile and in other similar working contexts.

Lastly, taking into account the important occupational mental health problems that Chile presents, and that the identified risk agents are linked to dysfunctional leadership, lack of social support, and work overload—all associated with situations of workplace bullying—it is suggested that the public policy of occupational health protection should provide measures to prevent, protect, and intervene against workplace bullying as a precursor to mental health problems. In this sense, our study adds relevant information to policy makers regarding the revision of these policies and the incorporation of workplace bullying and the attention to gender inequalities as essential factors in the health care of the working population.

## Figures and Tables

**Table 1:** Weighted distribution of the frequencies and percentages of exposure to workplace vulnerability according to sex.

Covariates	Women (%)^1^	F (*p*) ^2^	Men (%) ^1^	F (*p*) ^2^
WB	52 (16.8%)	22.80 ***	64 (16.3%)	13.20 ***
Psychological Distress	95 (30.8%)	49.30 ***	65 (16.5%)	19.00 ***
ISOSTRAIN	61 (21.6%)	29.90 ***	67 (17.8%)	43.80 ***
Effort–Rewards Imbalance	196 (65.4%)	95.40 ***	217 (58.9%)	66.30 ***
Low Job Satisfaction	127 (41.3%)	34.90 ***	136 (34.6%)	25.60 ***
Authoritarian Leadership	79 (25.8%)	20.90 ***	92 (23.3%)	18.10 ***
Economic Narrowness	126 (40.9%)	10.30 **	139 (35.7%)	26.40 ***
GSE (D and E)	84 (27.2%)	2.05	120 (30.5%)	0.44

^1^ The box counts of some categories are not integers. They have been rounded off to the nearest integers before testing for column proportions; ^2^ Measure of Rao–Scott association (F) and significance (*p*) codes: ** *p* < 0.01. *** *p* < 0.001. WB: Workplace Bullying; ISOSTRAIN: High job demands plus low decisional latitude and low social support in the workplace; GSE: Socioeconomic Groups.

**Table 2:** Associations between workplace vulnerability and risk of exposure to workplace violence and psychological distress, and between workplace violence and risk of exposure to psychological distress while controlling for workplace vulnerability in women and men.

	Females				Males			
	ORʳᵃʷ [95% CI]	OR^1^ [95% CI]	OR^2^ [95% CI]	OR^3^ [95% CI]	ORʳᵃʷ [95% CI]	OR^1^ [95% CI]	OR^2^ [95% CI]	OR^3^ [95% CI]
**Vul ➔ WB**								
	3.293 ***	1.972 *	2.212 **	3.504 ***	2.321 ***	1.429	1.564	2.229 **
	[1.98–5.49]	[1.07–3.64]	[1.30–3.76]	[2.10–5.86]	[1.46–3.69]	[0.87–2.35]	[0.95–2.58]	[1.38–3.61]
**Vul ➔ Distress**								
	4.161 ***	2.935 ***	3.219 ***	4.018 ***	2.952 ***	1.883 *	2.165 **	2.720 ***
	[2.74–6.31]	[1.84–4.69]	[2.09–4.97]	[2.62–6.17]	[1.78–4.89]	[1.08–3.30]	[1.28–3.67]	[1.61–4.59]
**WB ➔ Distress**								
	2.285 **	1.900 *	1.436	2.130 **	4.274 ***	2.925 **	2.393 *	4.014 ***
	[1.35–3.88]	[1.03–3.50]	[0.77–2.68]	[1.24–3.65]	[2.33–7.86]	[1.54–5.57]	[1.20–4.77]	[2.16–7.46]

➔: Logistic regression in which the variable of the left is the independent variable, and the variable at the right of the symbols is the dependent variable; 95% CI: 95% confidence interval; OR^raw^: Crude odds ratio; OR^1^ Controlling for psychosocial dimensions of work organization (Effort–Rewards Imbalance and ISOSTRAIN); OR^2^: Controlling for labor variables (Authoritarian Leadership and Job Satisfaction); OR^3^: Controlling for socioeconomic variables (Socioeconomic Group and Economic Narrowness); ^a^ Controlling for workplace vulnerability: Wald test significance codes (z); weighted variables: * *p* < 0.05. ** *p* < 0.01. *** *p* < 0.001; Vul = Workplace Vulnerability; Distress = Psychological Distress; WB = Workplace Bullying; W = women, M = men.

## References

[1] Einarsen S., Hoel H., Zapt D., Cooper C.L. (2011). Bullying and Harassment in the Workplace.

[2] Einarsen S. (2000). Harassment and bullying at work. Aggress. Violent Behav..

[3] Chapell D., Di Martino V. (2006). Violence at Work.

[4] Ariza-Montes J.A., Muniz R N.M., Leal-Rodríguez A.L., Leal-Millán A.G. (2014). Workplace bullying among managers: a multifactorial perspective and understanding. Int. J. Environ. Res. Public Health.

[5] Benach J., Julià M., Bolíbar M., Amable M., Vives A. (2018). Precarious employment, health, and quality of life. Violence and Abuse In and Around Organisations.

[6] Llosa-Fernández J.A., Menéndez-Espina S., Agulló-Tomás E., Rodríguez-Suárez J. (2018). Job insecurity and mental health:A meta-analytical review of the consequences of precarious work in clinical disorders. An. Psicol..

[7] Keim A.C., Landis R.S., Pierce C.A., Earnest D.R. (2014). Why do employees worry about their jobs? A meta-analytic review of predictors of job insecurity. J. Occup. Health Psychol..

[8] Cheng G.H.-L., Chan D.K.-S. (2008). Who Suffers More from Job Insecurity? A Meta-Analytic Review. Appl. Psychol..

[9] De Cuyper N., Baillien E., De Witte H. (2009). Job insecurity, perceived employability and targets’ and perpetrators’ experiences of workplace bullying. Work Stress.

[10] Shoss M.K. (2017). Job Insecurity: An Integrative Review and Agenda for Future Research. J. Manage..

[11] Hoel H., Salin D., Einarsen S., Hoel H., Cooper C. (2002). Organisational antecedents of workplace bullying. Bullying and Emotional Abuse in the Workplace.

[12] Djurkovic N., Noronha E., D’Cruz P. (2018). Workplace Bullying in Precarious Employment. Handbooks of Workplace Bullying, Emotional Abuse and Harassment.

[13] Einarsen S., Hauge L.J. (2006). Antecedents and consequences of workplace mobbing: a literature review. Revista Psicol. del Trab. y las Organ..

[14] Shin Y., Hur W.-M. (2019). When Do Service Employees Suffer More from Job Insecurity? The Moderating Role of Coworker and Customer Incivility. Int. J. Environ. Res. Public Health.

[15] Díaz X., Mauro A., Ansoleaga E., Toro J.P. (2017). Gender violence at work in Chile. An ignored field of study. Cienc. Trab..

[16] Milner A., King T., LaMontagne A.D., Bentley R., Kavanagh A. (2018). Men’s work, Women’s work, and mental health: A longitudinal investigation of the relationship between the gender composition of occupations and mental health. Soc. Sci. Med..

[17] Yoo G., Lee S. (2018). It Doesn’t End There: Workplace Bullying, Work-to-Family Conflict, and Employee Well-Being in Korea. Int. J. Environ. Res. Public Health.

[18] Messing K., Punnett L., Bond M., Alexanderson K., Pyle J., Zahm S., Wegman D., Stock S.R., de Grosbois S. (2003). Be the fairest of them all: Challenges and recommendations for the treatment of gender in occupational health research. Am. J. Ind. Med..

[19] Armstrong P., Messing K. (2014). Taking Gender into Account in Occupational Health Research: Continuing Tensions. Policy Pract. Heal. Saf..

[20] Puga I., Soto D. (2018). Social Capital and Women’s Labor Force Participation in Chile. Fem. Econ..

[21] Stecher A. (2014). Fairclough and language in New Capitalism: Analysis of the discursive dimensions of the world of labor. Psicoperspectivas. Individuo y Soc..

[22] Stecher A., Godoy L., Toro J.P. (2010). Supermarket work conditions and experiences: An exploration of labor flexibilization in Chilean Retail. Polis.

[23] Pérez-Franco J. (2016). New jobs, new risks. Chile and psychosocial risk factors in the workplace. Rev. Chil. Salud Pública.

[24] Rodriguez J.K., Gomez C.F. (2009). HRM in Chile: the impact of organisational culture. Empl. Relations.

[25] Darío Rodríguez M., René Ríos F. (2009). Paternalism at a crossroads: labour relations in Chile in transition. Empl. Relations.

[26] Vives-Vergara A., González-López F., Solar O., Bernales-Baksai P., González M.J., Benach J. (2017). Precarious employment in Chile: psychometric properties of the Chilean version of Employment Precariousness Scale in private sector workers. Cad. Saude Publica.

[27] Ansoleaga E., Gómez-Rubio C., Mauro A. (2015). Violencia laboral en América Latina: una revisión de la evidencia científica. Vertex.

[28] Vives A., Amable M., Ferrer M., Moncada S., Llorens C., Muntaner C., Benavides F.G., Benach J. (2010). The Employment Precariousness Scale (EPRES): psychometric properties of a new tool for epidemiological studies among waged and salaried workers. Occup. Environ. Med..

[29] Alvarado R., Pérez-Franco J., Saavedra N., Fuentealba C., Alarcón A., Marchetti N., Aranda W. (2012). Validation of a questionnaire for psychosocial risk assessment in the workplace in Chile. Rev. Med. Chil..

[30] Vergara A.V., Fabra U.P. The Employment Precariousness Scale ( EPRES ): Origin, Development and Applications 2012. https://www.google.com.hk/url?sa=t&rct=j&q=&esrc=s&source=web&cd=1&ved=2ahUKEwjp786y46LlAhWFMt4KHRiFDREQFjAAegQIABAC&url=https%3A%2F%2Fwww.etui.org%2Fcontent%2Fdownload%2F5753%2F56205%2Ffile%2FThe%2BEmployment%2BPrecariousness%2BScale.pdf&usg=AOvVaw3UB0v6zmlg4gkqeMxn9Eqn.

[31] Einarsen S., Raknes B.I. (1997). Harassment in the Workplace and the Victimization of Men. Violence Vict..

[32] Gonzalez D., Graña J.L. (2009). Workplace bullying: Prevalence and descriptive analysis in a multiocupational sample. Psicothema.

[33] Mikkelsen E.G., Einarsen S. (2001). Bullying in Danish work-life: Prevalence and health correlates. Eur. J. Work Organ. Psychol..

[34] Ansoleaga E., Montaño R., Michel V. (2013). Validation of two complementary instruments for measuring work stress in Chilean workers. Scand. J. Organ. Psychol..

[35] Karasek R., Brisson C., Kawakami N., Houtman I., Bongers P., Amick B. (1998). The Job Content Questionnaire (JCQ): an instrument for internationally comparative assessments of psychosocial job characteristics. J. Occup. Health Psychol..

[36] Siegrist J., Wege N., Pühlhofer F., Wahrendorf M. (2009). A short generic measure of work stress in the era of globalization: effort–reward imbalance. Int. Arch. Occup. Environ. Health.

[37] Judge T.A., Parker S., Colbert A.E., Heller D., ILIES R. Job Satisfaction: A Cross-Cultural Review. Handbook of Industrial, Work & Organizational Psychology-Volume 2: Organizational Psychology.

[38] Skogstad A., Aasland M.S., Nielsen M.B., Hetland J., Matthiesen S.B., Einarsen S. (2014). The Relative Effects of Constructive, Laissez-Faire, and Tyrannical Leadership on Subordinate Job Satisfaction. Z. Psychol..

[39] Aasland M.S., Skogstad A., Notelaers G., Nielsen M.B., Einarsen S. (2009). The Prevalence of Destructive Leadership Behaviour. Br. J. Manag..

[40] Leymann H. (1990). Mobbing and psychological terror at workplaces. Violence Vict..

[41] Scott A. (2007). Rao-Scott Corrections and Their Impact; Auckland. http://www.asasrms.org/Proceedings/y2007/Files/JSM2007-000874.pdf.

[42] Agresti A. (2013). Categorical Data Analysis.

[43] (2011). European Foundation for the Improvement of Living and Working Conditions Rise in Reported Cases of Bullying and Violence at Work. Dublin. https://www.eurofound.europa.eu/sites/default/files/ef_files/ewco/surveyreports/DK1108019D/DK1108019D.pdf.

[44] Manuel S.K., Howansky K., Chaney K.E., Sanchez D.T. (2017). No Rest for the Stigmatized: A Model of Organizational Health and Workplace Sexism (OHWS). Sex Roles.

[45] De Puy J., Romain-Glassey N., Gut M., Wild P., Pascal W., Mangin P., Danuser B. (2015). Clinically assessed consequences of workplace physical violence. Int. Arch. Occup. Environ. Health.

[46] Finne L.B., Knardahl S., Lau B. (2011). Workplace bullying and mental distress—A prospective study of Norwegian employees. Scand. J. Work. Environ. Health.

[47] Attell B.K., Kummerow Brown K., Treiber L.A. (2017). Workplace bullying, perceived job stressors, and psychological distress: Gender and race differences in the stress process. Soc. Sci. Res..

[48] Milner A., Scovelle A., King T., Marck C., McAllister A., Kavanagh A., Shields M., Török E., O’Neil A. (2019). Gendered Working Environments as a Determinant of Mental Health Inequalities: A Protocol for a Systematic Review. Int. J. Environ. Res. Public Health.

[49] Ansoleaga E., Díaz X., Mauro A. (2016). Psychosocial risks, quality of employment, and workplace stress in Chilean wage-earning workers: A gender perspective. Cad. Saude Publica.

[50] García Johnson C.P., Otto K. (2019). Better Together: A Model for Women and LGBTQ Equality in the Workplace. Front. Psychol..

[51] Ansoleaga E. Organizational Dimensions of Violence at Work in Chile: A Study in Three Economic Sectors Addressing Occupational and Gender Differences. https://iawbh.org/resources/Documents/IAWBH_Newsletter_No_30_2016_December.pdf.

[52] Roscigno V.J., Hodson R., Lopez S.H. (2009). Workplace incivilities: the role of interest conflicts, social closure and organizational chaos. Work. Employ. Soc..

[53] Gideon J., Moreno E.A. (2016). Gendered work violence issues and mental health among Chilean women workers. Handbook on Gender and Health.

[54] Ansoleaga E., Diaz X., Mauro A., Gideon J. (2015). Gendered Work Violence Issues and Mental Health among Chilean Women Workers. Gender and Health.

[55] Lanthier S., Bielecky A., Smith P.M. (2018). Examining Risk of Workplace Violence in Canada: A Sex/Gender-Based Analysis. Ann. Work Expo. Heal..

[56] Gardner D., O’Driscoll M., Cooper-Thomas H., Roche M., Bentley T., Catley B., Teo S., Trenberth L. (2016). Predictors of Workplace Bullying and Cyber-Bullying in New Zealand. Int. J. Environ. Res. Public Health.

[57] Nielsen M.B., Indregard A.-M.R., Overland S. (2016). Workplace bullying and sickness absence: a systematic review and meta-analysis of the research literature. Scand. J. Work. Environ. Health.

[58] Bureau J.S., Gagné M., Morin A.J.S., Mageau G.A. (2017). Transformational Leadership and Incivility: A Multilevel and Longitudinal Test. J. Interpers. Violence.

[59] Glambek M., Skogstad A., Einarsen S. (2018). Workplace bullying, the development of job insecurity and the role of laissez-faire leadership: A two-wave moderated mediation study. Work Stress.

[60] Tourangeau R., Yan T. (2007). Sensitive questions in surveys. Psychol. Bull..

[61] Geoffroy M., Chamberland L. (2015). Mental health implications of workplace discrimination against sexual and gender minorities: A literature review. Sante Ment. Que..

[62] Straiton M.L., Aambø A.K., Johansen R. (2019). Perceived discrimination, health and mental health among immigrants in Norway: the role of moderating factors. BMC Public Health.

